# “Problems you can live with” versus emergencies: how community members in rural Ethiopia contend with conditions requiring surgery

**DOI:** 10.1186/s12913-024-10620-0

**Published:** 2024-02-16

**Authors:** Hanna Negussie, Medhanit Getachew, Andualem Deneke, Amezene Tadesse, Ahmed Abdella, Martin Prince, Andrew Leather, Charlotte Hanlon, Chris Willott, Rosie Mayston

**Affiliations:** 1 Center for Innovative Drug Development and Therapeutic Trials for Africa (CDT- Africa), College of Health Sciences, Addis Ababa University, P.O. Box 9086, Addis Ababa, Ethiopia; 2 Department of Surgery, School of Medicine, College of Health Sciences, Addis Ababa University, Addis Ababa, Ethiopia; 3 Department of Obstetrics and Gynaecology, School of Medicine, College of Health Sciences, Addis Ababa University, Addis Ababa, Ethiopia; 4 King’s Global Health Institute, King’s College London, London, UK; 5 King’s Centre for Global Health and Health Partnerships, School of Life Course and Population Sciences, King’s College London, London, UK; 6 Centre for Global Mental Health, Health Service and Population Research Department, Institute of Psychiatry, Psychology and Neuroscience, King’s College London, London, UK; 7 Global Health & Social Medicine, King’s College London, London, UK

**Keywords:** Global surgery, Explanatory models, Help-seeking behaviour, Qualitative research, Ethiopia

## Abstract

**Background:**

98% of people with surgical conditions living in low- and middle-income countries (LMICs) do not receive safe, timely and affordable surgical and anesthesia care. Research exploring barriers to receiving care has tended to be narrow in focus, often facility-based and ignoring the community beliefs, experiences and behaviours that will be an essential component of closing the gap in surgical care. Using qualitative methods, we captured diverse community perspectives in rural Ethiopia: exploring beliefs, perceptions, knowledge and experiences related to surgical conditions, with the overall aim of (re)constructing explanatory models.

**Methods:**

Our study was nested within a community-based survey of surgical conditions conducted in the Butajira Health and Demographic Surveillance Site, southern Ethiopia, and a follow-up study of people accessing surgical care in two local hospitals. We carried out 24 semi-structured interviews. Participants were community members who needed but did/did not access surgical care, community-based healthcare workers and traditional bone-setters. Interviews were conducted in Amharic, audio-recorded, transcribed, and translated into English. We initially carried out thematic analysis and we recognized that emerging themes were aligned with Kleinman’s explanatory models framework and decided to use this to guide the final stages of analysis.

**Results:**

We found that community members primarily understood surgical conditions according to severity. We identified two categories: conditions you could live with and those which required urgent care, with the latter indicating a clear and direct path to surgical care whilst the former was associated with a longer, more complex and experimental pattern of help-seeking. Fear of surgery and poverty disrupted help-seeking, whilst community narratives based on individual experiences fed into the body of knowledge people used to inform decisions about care.

**Conclusions:**

We found explanatory models to be flexible, responsive to new evidence about what might work best in the context of limited community resources. Our findings have important implications for future research and policy, suggesting that community-level barriers have the potential to be responsive to carefully designed interventions which take account of local knowledge and beliefs.

**Supplementary Information:**

The online version contains supplementary material available at 10.1186/s12913-024-10620-0.

## Introduction

Surgical cases are estimated to account for 28–32% of the overall burden of disease globally. In low-and middle-income countries (LMICs) [1], 14.2% of the overall burden of disease is attributed to conditions needing basic surgical interventions such as maternal and neonatal disorders, abdominal conditions and injuries [2]. Despite the population burden, around 67% of the world’s population do not have access to surgical care. This is disproportionately higher in LMICs where 98% of the population lack access to safe, affordable and timely surgical care [3]. It is evident that a gap in infrastructure [4], lack of facilities, equipment, anesthesia, critical care provision and availability of specialists are major supply-side barriers to accessing surgical care in LMICs [5]. However, community knowledge and beliefs, in some cases linked to perceived infrastructural weaknesses, as well as to household economic constraints, are an important driver of help-seeking for conditions requiring surgical care [5, 6].

There is a small but growing body of qualitative evidence that documents the ways in which community members in various sub-Saharan African (SSA) countries conceptualize and approach health problems where there is a need for surgery. Fear of surgery has been found to inhibit access to surgical care [7, 8], originating from a combination of lack of knowledge about surgery and its efficacy and cultural beliefs about surgical procedures. For example, a focus group study carried out in a community setting in Sierra Leone found that participants feared being ‘half human’ or incomplete after surgery [7]. In rural South Africa, older people who had severely impaired vision due to operable cataract reported fear of surgery or a belief that blindness in old age was inevitable or irreversible [9]. Costs of care, in terms of treatment, transportation and lost opportunities for income generation have been found to be prohibitive. As a result, surgical care pathways often incorporate multiple providers and different treatment modalities, which may be shaped by preference, availability and accessibility [6]. For example, in Kenya, women with obstetric fistula reported that they tried different interventions including home remedies, divine or traditional medicine, prayer, private clinics or stayed home even after primarily seeking care from a hospital [10]. In a qualitative study conducted among surgeons in Ethiopia, in addition to financial constraints and cultural beliefs, a lack of community awareness about surgical conditions and where to access surgical care were felt to be the causes of delays in help-seeking, with patients only coming to health facilities only after their condition had become severe [11].

For those experiencing them, surgical conditions embody much more than the specific biomedical problem that surgery is designed to resolve. Individual understanding of a condition and ideas about what to do about it are influenced by a complex mix of social and cultural factors as well as individual psychology and experience. With the exception of obstetric research, surgical research exploring barriers to receiving care has tended to be narrow- often facility-based and not focused on help-seeking in the community [12]. And yet, understanding the whole surgical ecosystem, including knowledge of the community beliefs and behaviours will be essential to closing the gap in surgical care [13]. Whereas previous studies have explored perceptions of individual surgical conditions, in our study we sought to understand the ways in which people recognized and dealt with different types of problems where surgery was indicated. Explanatory models have been most commonly used in mental health research as a tool to help develop an understanding people’s ideas about their illness: its aetiology, the onset of symptoms, pathophysiology, course of illness and treatment [14]. In this study, we aimed to capture diverse community perspectives to explore beliefs, perceptions, knowledge and experiences related to conditions where clinicians would recommend surgery in rural Ethiopia, with the aim of describing the community’s explanatory model(s) for surgical conditions.

## Methods

### Design

The study design was qualitative, nested within the surgery work package of ASSET, a health systems strengthening research programme working across four SSA African countries (Ethiopia, Sierra Leone, South Africa, Zimbabwe) and three platforms of care (surgery, integrated primary care and maternal and newborn care) [15]. One of the overall aims of ASSET was to develop effective health system strengthening interventions to support the translation of clinical evidence into delivery of integrated continuing care at scale across healthcare platforms for surgical and dental care in Ethiopia. This qualitative study/design was used to explore/understand the unmet needs of people with surgical conditions who do, and do not, access surgical care, the impact of unmet needs for surgical care. In addition, this study aimed to understand how community members in rural Ethiopia deal with surgical conditions, explore the barriers and facilitators to improving access to quality surgical care, and what are the patterns of help-seeking. These questions would help us to better understand the problems that people face in accessing surgical care at the community level. This knowledge would then help us to design interventions to strengthen the health system and reduce delays in accessing care, which would ultimately reduce delays in accessing care and improve help-seeking. The ASSET research team was multidisciplinary and research questions, the study design and methods were co-designed with policymakers and healthcare professionals, including local surgeons. The research team has a longstanding partnership with the local community in the districts the study was conducted. Prior to starting the qualitative study, theory of change workshops that included community members, local health professionals and administrators from the district, helped to refine areas to explore within the topic guide.’

### Context

This study was conducted in neighboring sites: Butajira town, Meskan, Misrak Meskan, Debub Sodo and Sodo districts, which are found in the Gurage Zone, Southern Nations, Nationalities and People’s region (SNNPR), Ethiopia. Guragigna is spoken by most of the population as their mother tongue, whereas Amharic serves as the official language of the region. The area is predominantly rural, with an economy based upon subsistence farming and small trade, characteristic of the majority of Ethiopia. Butajira town, Meskan and Misrak Meskan districts are located 130 km whereas Debub Sodo and Sodo districts are located 100 km from Addis Ababa, the capital of Ethiopia, respectively. The total population of Butajira town, Meskan, Misrak Meskan, Debub Sodo and Sodo districts was projected to be 344,294 in 2014. Most of the population in Butajira town are Muslim, whereas in Sodo district, most of the population is Ethiopian Orthodox Christian (97%). Butajira hosts a health and demographic surveillance site (HDSS) which has been functioning since 1987 [16]. In Butajira town, surgical care was available in a general hospital. At the time of data collection, the hospital had two surgeons and two Integrated Emergency Surgical Officers (IESOs) whose role is to manage emergency obstetric, gynecological and surgical cases [17]. In Sodo district, surgical care was delivered in a primary hospital where there were only two IESOs.

### Participants

The study participants comprised community members with probable surgical conditions who had, or had not, subsequently accessed care as well as community health workers (known as Health Extension Workers (HEWs) in Ethiopia) and traditional bone setters, all of whom are known to play an important role within the pathway to care for surgical conditions. Purposive sampling was used with two ASSET quantitative surveys about conditions requiring surgery as the sampling frames for the study. Firstly, 182 participants who had accessed surgical care were identified through a facility based surgical cohort study, carried out at Butajira (*n* = 166) and Buee (*n* = 16) hospitals. This included participants who had accessed emergency or elective surgical care, for example: cesarean section and appendectomy. From this cohort study, six participants were selected and included in this study. Secondly, nine participants with a probable or definite surgical condition who had not accessed surgical care and one participant who had accessed surgical care were selected from a larger surgical community survey which was conducted in the Butajira Health and Demographic Surveillance Site (*N* = 1061 participants).

The presence of a surgical condition was determined by surgical residents after examination during the survey. We aimed to recruit participants with a variety of surgical conditions (see Table 1) and diversity in terms of the background characteristics we thought were most likely to shape experiences. We included a mixture of participants from the urban area close to the Butajira town hospital, rural villages, as well as a balance by gender and educational level (see Table 1). We purposively selected five HEWs and three bone setters to be included in this study. They were selected based on their experience and being well known within the community.

### Data collection

Topic guides were developed for semi-structured interviews, adapted for the different stakeholder groups. Interviews explored awareness about surgical conditions, help-seeking behavior, pathways of care, including barriers and facilitators to accessing surgical care, the experience of surgical care and the lasting impact of the condition and what kind of non-biomedical interventions are available and accessed. There were four interviewers. Interviewers were all research assistants with a master’s degree who had prior experience of conducting semi-structured interviews in health research.

Interviews were conducted in Amharic and audio-recorded. Some of the interviews with people who had an identified surgical condition took place in their homes and the remainder took place in health centers, according to the preference of interviewees. The interviews with HEWs and bone setters were conducted in the project offices in Sodo and Butajira. The median interview length was 19.5 min, with a minimum of 8.3 and maximum of 54.3 min.

### Analysis

Interviews were first transcribed in Amharic and then translated into English and uploaded into OpenCode 4.02 software for analysis [18]. Interviews were translated to English as the senior researchers mentoring the lead author were non-Amharic speakers. To ensure specific and local meanings were retained, where local idioms and phrases were ambiguous or difficult to translate, the Amharic was maintained in transcripts alongside the English, discussed between members of the research team until an appropriate translation could be agreed. The constitution of the team helped to ensure that the research maintained a delicate balance between external validity and local credibility. Local researchers, bilingual in English and Amharic were also bi-cultural- trained in biomedical and social science but culturally embedded in Ethiopia worked alongside a culturally sensitized non-Amharic speaking senior researcher from the UK as part of a longstanding research partnership between Addis Ababa University and King’s College London. We discussed each stage of the analysis, together, explicitly recognizing our different positionalities and the implications of these for our work. To check the equivalence of meaning of translations the Amharic-speaking authors checked the Amharic transcripts, highlighting any challenging areas and referring to the audios where necessary before discussing and resolving any ambiguous/contested translations.

Our approach to analysis evolved over time, moving beyond the original study objectives to describe unmet needs for surgery and barriers and facilitators to accessing surgical care, in response to data that was both richer and thicker than anticipated. Our initial plan was to carry out inductive thematic analysis. Accordingly, two members of the research team (HN, MG) read and re-read transcripts to familiarize themselves with the data and independently carried out line-by-line coding of three randomly selected transcripts. Excerpts of text perceived to be relevant to research questions were assigned defined codes. HN and MG met twice to compare and refine codes and coding, first after coding two transcripts and then after coding a third transcript, developing a common codebook through discussion of each code definition and assignment. RM reviewed the codebook and met with HN and MD to finalise areas of uncertainty (potential overlapping codes, poorly defined codes). HN and MD then independently coded the remaining transcripts based on the codebook, identifying, discussing and defining new codes where necessary.

Next, coded text was examined and possible links between different codes were considered. HN, MG and RM met to discuss possible clusters of codes and the rationale for connections, as well as the potential themes that emerged from this work. Themes were then reviewed, with input from CW, and checked against data to ensure they represented the experiences narrated in interviews. Quotes from a range of participants were selected to illustrate these themes. Themes which were not well-represented were dropped. At this stage, we recognized that emerging themes were aligned with Kleinman’s explanatory models framework and decided to use this to guide the final stages of analysis- enabling us to go beyond the stated study objectives to explore causal models for health-seeking behaviours related to conditions indicating surgery. Accordingly, informed by framework analysis [14], we carried out within-case analysis examining the links in individual interviews between: participants’ description of the problem, their understanding of cause and course, health-seeking actions, reasons for these and fears/risks associated with actions/inaction. Finally, we compared these individual explanatory models, paying particular attention to the commonalities and differences in the beliefs, experiences and perceptions that participants cited as motivations and rationales for their (in)actions. This enabled us to construct the community explanatory model described in the text which details how particular categories of problem were associated with common patterns of health-seeking, which, in turn, were shaped by shared experiences and beliefs.

### Researcher positionality

The researchers involved in this study are Ethiopian clinicians, social epidemiologists or social scientists based in universities who share an interest in health system strengthening to improve population health outcomes. The approach and topic guides were informed by an awareness of the need to ensure respondents did not perceive an expectation to endorse biomedical paradigms. Potential power differentials existed between our urban, educated interviewers and rural interviewees with limited formal education. Interviewers were trained to consider the respondent as the expert and made efforts to make the interviewee comfortable to express themselves freely. Team discussions were held to bring different perspectives into the analysis.

### Ethical considerations

Ethical clearance was obtained from the Institutional Review Board (IRB) of the College of Health Sciences of Addis Ababa University (026/18/PSY; 16th May 2018) and King’s College London (RESCM-17/18-6144; 18th August 2018). Written informed consent was obtained from each participant. For participants who were not able to read and write, the interviewer read out the information sheet in front of another person, known to the participant, who was literate and able to confirm that full and accurate information was given. A witness statement was included in the informed consent form for such cases in addition to the participant’s consent, which was indicated by a thumb print. For the participant who was aged less than 16 years old, informed consent was obtained from the parent and the interview was also conducted with the parent.

**Table 1:** Socio-demographics of participants

Characteristics	Frequency (*N* = 24)	Percent
**Sex**		
Male	8	33.3
Female	16	66.7
**Type of respondent**		
Patient who accessed surgical care	7	29.2
Patient who did not access surgical care	9	37.5
Health extension worker	5	20.8
Traditional bone setter	3	12.5
**Age (in years)**		
10–20	4	16.7
21–30	11	45.8
31–40	4	16.7
41–50	0	0
51–60	2	8.3
61–70	2	8.3
71–80	1	4.2
**Educational level**		
No formal education	1	4.5
Primary only	8	36.4
Secondary only	5	22.7
Above secondary	8	36.4
**Religious affiliation**		
Orthodox Christian	10	41.7
Muslim	10	41.7
Protestant Christian	4	16.6
**Types of surgical conditions**		
**Non-emergency**		
Eye problem	1	6.3
Gall stones	1	6.3
Hypertrophic scar	1	6.3
Kidney stones	2	12.5
Lipoma	1	6.3
Tonsillitis	1	6.3
**Emergency**		
Appendicitis	3	18.7
Childbirth complications	3	18.7
Extremity fracture	1	6.3
Right hip joint trauma	1	6.3
Shoulder dislocation	1	6.3

## Results

We carried out 24 semi-structured interviews in total. Participants included those who had surgical conditions, both those who accessed surgical care (*n* = 7) and those who did not access surgical care (*n* = 9). We also included community HEWs (*n* = 5) and traditional bone setters (*n* = 3) who live within the community. See Table 1 for participant characteristics. We present the results in two main themes: (1)“problems you can live with” which describes how community members deal with surgical conditions that are perceived not to require urgent intervention and (2) those that are classified as emergencies, which require urgent surgical care.

### Problems you can live with

Surgical conditions that do not trigger immediate help-seeking by community members are presented in three sub-themes: addressing bearable ailments; knowledge, fear and community narratives; and counting the costs of care.

### Addressing bearable ailments

Conditions where symptoms appeared gradually and which were considered tolerable when the ailment started did not trigger immediate help-seeking at a biomedical facility. Rather, participants reported trying self-help or traditional remedies first, visiting a health facility only when symptoms worsened and became unbearable.

*“When it first started, I washed my eyes using soap and other traditional treatments like, herbs and I got better. I thought it was an allergy but it wasn’t…it was even getting worse. Then, we went to Dukuman [NGO treatment facility]”* [ID SCS04, 65 year old female community member with eye problem].

Whilst treatment preferences varied, experimenting with different types of health facilities and repeated visits was common when conditions did not show improvement.

*“First I went to one private clinic… Then I went to another clinic but I didn’t get any improvement. Then, I went to Werabe Hospital [public hospital] and they told me that, “you don’t have that much problem and it is just something not serious”.… And I went there, to Werabe hospital once more.. then, they checked me with ultrasound… and they gave me a medication and sent me back home. But, I got sick again and I went to another private clinic…”* [ID SCS03, 17 year old female community member with kidney stones].

Help-seeking was described as a response to symptoms. When these ebbed and flowed, help-seeking followed the same pattern:

*“Since I was getting sick now and again, I felt tired of it and I was undecided to go or not to go. I may think of going but I don’t really go there when I feel like I am getting better. I decide to go when I feel sick but I stop going there when I get better”* [ID SCS03, 17 year old female community member with kidney stones].

As help-seeking largely depended on perceived severity of symptoms, there were community members who lived with surgical conditions without seeking care, sometimes for extended periods of time, whilst they considered whether to attend the hospital for care.

### Knowledge, fear and community narratives

Respondents acknowledged that knowledge of surgery, its scope, application and benefits for various conditions was only recently acquired and was incomplete, making it difficult for community members to identify those conditions suitable for surgical care. This knowledge gap was felt to compound the effects of fear and further inhibit motivation to seek surgical care.

This knowledge gap also seemed to be present among healthcare workers. Community members provided examples of conditions where surgical teams had identified a need for surgical care but where they were later advised by other healthcare professionals that their problem could be addressed via non-surgical means.

Not all surgical procedures or facilities were viewed equally. Attributes of a particular facility might help to enhance that facility’s reputation for success. For example, community members were reported to have confidence in one facility which is known to provide effective, safe surgical care for specific conditions and is run by foreigners:

*“many people are getting their eye vision back. People come from Jimma, they also come from Sidamo, Addis Ababa and from many places… they do work very much. Plus it is American’s organization*” [ID SCS04, 65 year old female community member with eye problem].

Fear of surgical procedures was also identified by healthcare workers and community members as a key reason for avoiding surgical care.

*“They are scared. There was this woman who had a uterine prolapse. We tried to get her to do surgery but she refused. Even though she knows there are five other women who went through the same procedure and they are fine. Another woman who has goiter, we took her to a place that does surgery but she refused. She came back without the surgery.”* [ID S08, 30 year old female HEW].

Healthcare workers described the behaviours adopted by patients to delay or evade surgical care:

*“I also know another woman who always changes her appointment. There is also another man who has a tumor however he changes his appointment and tricks his doctors.”* [ID S13, 28 year old female HEW].

However, HEWs explained that many people in the community are starting to accept surgical care due to positive narratives circulating in the community. Community members whose condition improved after surgical care informed others of the results and this is increasing the acceptance to surgical care among the community.

*“They are accepting it well. The one who got healed informs the others and it goes on; and they come to me [to get referral] and get treated.”* [ID S11, 28 year old female HEW].

They also highlighted examples of community members who had been persuaded to undergo surgery despite their fears and experienced a positive outcome which then changed their perception of surgery.

*“For example there was this woman who refused the surgery but after her family convinced her she was treated. And she always says ‘what was I thinking?!.’”* [ID S13, 28 year old female HEW].

For some, negative narratives shared in the community of others who had undergone surgery continued to play a role in shaping distrust of surgical care.

*“They [community members] do talk among themselves a lot. If one of them said that they did something to her or the treatment did something to her, they will transfer the information with each other… yes even if people wanted to do it they will get scared. And they won’t be willing.”* [ID S08, 30 year old female HEW].

Alongside fear, lack of knowledge and concerns about quality of care, there was a common belief among community members that surgery was not for them. This belief was held alongside an innate preference for spiritual services, which were perceived to be particularly well suited to specific conditions, such as kidney stones.

*“Regarding kidney stone most people think they will be healed by drinking water, I mean holy water [which is water considered to be blessed and believed to heal the sick and also cast out demons among Christians]. They say they will drink water. Most say like that. But some say they had an appointment in Butajira but didn’t go since they got cured by drinking the holy water. They said it went away and they did not do the surgery on their appointment.”* [ID S09, 28 year old female HEW].

### Counting the costs of care

Low household income meant that most community members felt they could not afford to access surgical care for problems that they felt they could endure. Costs included medical fees, transportation costs and lost opportunities for income generation due to the time taken for travel:

*“Well I think it might be economical problem. For example, they might not have cash in their hand. Or they might live far away. There might be transportation problems. They might hope that they will get better tomorrow or the day after instead of going all that way. So, I think what prevents them from not going to health facilities as soon as they get sick is that they may not have money to pay for transport and medical bills.”* [ID S08, 30 year old female HEW].

Household responsibilities were raised as a factor in delaying care, particularly for women.

*“And even when she is having surgery here there is no one who will manage her home. She can’t leave her children”.* [ID S08, 30 year old female HEW]

### Emergency conditions

For sudden onset and severe conditions, the individual, family and community response differed depending on whether it was a maternal emergency, abdominal pain or an injury. Thus, the results for emergency conditions are presented as separate sub-themes for each surgical condition.

### Ensuring safe delivery

Surgical intervention for birth related complications was found to be accepted by women despite their residual fear of surgery. Health care facilities were reportedly the first choice of care for pregnant women. All women who had surgery to deliver a baby reported that they directly went to a hospital where they had received antenatal care either when their labor started or when amniotic fluid started leaking. Two of the women stated that they found out they were going to have surgery moments before the surgery was undertaken. For the most part, it appears that health care providers made decisions about women’s care without consultation with the women themselves:

*“Then, one of the doctors called me and told me to get inside…I didn’t know that I was going to give birth at that time but after I got inside, they secured an IV fluid and prepared other things and they said, ‘you are going to have a surgery’. But I didn’t have any sign of labor at that time.”* [ID SCS11, 25 year old female community member with childbirth related complication].

Women reported fear of undergoing surgery and its consequences but nonetheless, they agreed to go ahead with it. Positive narratives about the surgery and its outcome encouraged them to be willing, despite their fear.

*“I also get information from other people who did the surgery before. There are people who had the surgery before. So, I also get lesson from them. It is from family as well as from friends. There was a woman who gave birth at the same time with me. There is also a woman who works in the hospital and gave birth two months after I give birth. She gave birth by surgery. Since she knows how it is, she used to tell us that surgery is easy. I used to have fear but you face what you fear, right?”* [ID SCS14, 20 year old female community member with child birth related complication].

Recognition that a woman might need to have surgery to prevent birth complications and awareness that surgical services were available prompted women to seek care early at the hospital.

However, participants mentioned that some members of the community still encouraged women to go to a traditional bone setter for birth related complications. Women who were aware of surgical interventions for these complications were less influenced by such narratives and sought care from health facilities:

*“there are people who go to wegesha* [bone setter] *… It was other people who told me to go to wegesha. But I said I am not going to go and it would be corrected on its own. They [clinicians in the health facility] would have told me if I had to go another facility. So, I kept waiting. Then they did my surgery on its time.”* [ID SCS14, 20 year old female community member with child birth related complication].

Despite the fear and having to undergo surgery with minimal explanation from health care providers, participants reported they were satisfied with the overall service they received.

### Severe abdominal pain/appendicitis

Traditional remedies were reportedly the popular choice for severe abdominal pain in the past. However, the HEWs reported that nowadays formal health care facilities were preferred by community members. There were still differences in help-seeking behaviors within the community; some went directly to health facilities and others preferred to visit traditional healers or to try home remedies as an initial response. The main factors influencing these decisions include severity of pain, awareness created by community health workers, positive narratives within the community, lack of knowledge about the conditions and economic capacity.

Help-seeking in health facilities was reported to be growing for such conditions since HEWs are playing a role in educating community members about the harms of some traditional healing methods used to treat such conditions.

“*We will give them advice on that it’s a harmful tradition to cauterize a child’s abdomen. And since the child’s condition is treatable they should take him to health facilities. And after we tell them they usually take their children there.*” [ID S08, 30 year old female HEW].

But still, worsening severity of pain was the main driver for people with severe abdominal pain to seek care from a health facility.

*“They may stay at home hoping it will get better. But if it gets worse and when they can’t handle it [the pain], they will go to a health facility.”* [ID S08, 30 year old female HEW].

For such conditions, there were narratives where the experiences of community members stood in place of knowledge about biomedical causes and available treatment, supporting rapid decision-making and help-seeking.

*“At that time I had not that much knowledge. But I have heard people talking about other people who had this kind of disease and went to hospital and got healed. I heard that the treatment helped them very much and that they get well because they went to health facility.… Then when this happened to me the thought just came to my mind and I said ‘I am feeling sick and let me go to hospital immediately’ and then came to hospital.”* [ID SCS13, 56 year old male community member with appendicitis].

At the same time, in some narratives lack of knowledge about the causes of severe abdominal pain and available treatment was also identified as a factor in delayed help-seeking at health facilities. In addition, economic capacity played in to the decision of whether and when to seek care from a health facility, namely, the ability to pay for expected costs of treatment and transport. Because appendicitis is an acute condition that significantly disrupted people’s ability to carry out normal activities, many community members nonetheless often sought help from a health facility even if they had to borrow the money from others to meet the costs of treatment. However, in some cases, lack of funds caused people to cut short their stay in hospital:

*“we decided to get out. Because, they said, “you need to pay additional money quickly; you can’t stay here tonight if you can’t pay”. We borrowed the money from different places and we paid two thousand birr and we got out.”* [ID SCS15, 40 year old male community member with appendicitis].

### Accidental injuries

For injuries sustained in accidents, the decision to seek help and the type of treatment modality depended on the type of accident and the injury sustained. Most HEWs indicated that those involved in accidents caused by vehicles including cars, motorbike and *bajaj* (three wheelers) generally sought care from health facilities immediately and were taken by others to a health facility without delay. However, even then, lack of transportation and economic hardship sometimes contributed to delay in accessing care from a health facility after being involved in an accident, despite the potential severity of injury.

*“It is the transportation problem. They don’t get transportation right away. And if they do not have money, even if they bleed, they wouldn’t go to health facility.”* [ID S12, 27 year old female HEW].

In addition, one HEW stated the type of the treatment community members decide to seek depends on the severity of the pain after the accident.

*“when there is fracture, sometimes they go to wegesha [traditional bone setter] and get massage… when they face car accident, they do not immediately go to health facility. They wrap cloth around the damage to save their life… They do not know that much about infection and they have something they do culturally.… [when it is car accident or fracture, for example] they relate it with the pain. If the pain is slight, they have problem [they don’t seek care immediately]. But if the pain is severe, there is a chance that they could go [to a health facility] immediately.”* [ID S12, 27 year old female HEW].

This was reiterated by bone setters who reported that some people in such accidents seek care from them:

*“So, when it is a car accident, when they are hit in the head and when they are in a life or death situations, they take them to health facilities… And some will panic and prefer to go to bone setters. Especially when it is a shoulder dislocation.”* [ID S14, 40 year old male bone setter].

For accidental injuries, caused other than by vehicle accidents, resulting in dislocations, broken legs, sprains including fractures, it was indicated that bone setters were the first choice of care among community members. HEWs highlighted that bone setters are influential in the community with good reputation and are known to handle such cases. Moreover, the bone setters are also known to refer cases to health facilities for treatment if they felt the condition of the individual was outside of the scope of their expertise. This was also restated by the bone setters:

*“If I feel like it is beyond my capacity I refuse to do it because if you tie it wrongly it might cause amputation. My friend’s son got fractured while playing football and he couldn’t access me and went to somebody else and his legs ended up amputated. You see there are blood vessels around and when you fix the fracture if it is too tight it might stop circulation and lead to infection. And once this happens it will be gangrenous.”* [ID S14, 40 year old male bone setter].

Nevertheless, for some community members it was reasons such as economic capacity, lack of knowledge about traumatic injuries requiring medical care and also lack of availability of a nearby health facility providing surgical care which led them to seek care from a bone setter.

*“I mean they know that they will have to go to Wolayita [where there is a health facility which provides surgical care requiring orthopedic surgeon], and since they can’t afford to go to health institution there, they will say that they will just go to traditional bone settler.”* [ID S13, 28 year old female HEW].

For most accidental injuries, people sought help immediately as the pain was usually unbearable. However, there are community members who delayed accessing any type of care for some traumatic injuries where the pain worsens through time.

*“In the beginning, the pain was on and off type and I was relieved when I got rest. That is the reason that I stayed for a year. When it became worse, I went to the health facility two times.”* [ID SCS09, 40 year old female community member with right hip joint trauma].

## Discussion

Our findings have enabled us to construct an explanatory model which describes how people understand and experience surgical conditions in rural Ethiopia and the factors that influence decisions about help-seeking. This work represents an important contribution to the small body of evidence which seeks to understand the community-level influences on help-seeking: a critical but poorly understood component of the surgical ecosystem and a key constituent of closing the gap in access to safe, affordable care. One of the key strengths of our study is the inclusion of people with confirmed surgical conditions who had not accessed care, an important group who are difficult to reach and whom we identified by using a large community survey as our sampling frame.

As has been found in other LMICs, in our setting, community members afflicted by surgical conditions primarily understood these according to severity [19–22]. Severity was defined by two related constructs- whether the individual’s condition was perceived to be life-threatening and the severity of pain. Importantly, our findings show help-seeking was a dynamic process, with individuals responding to changes (or lack of change) in severity and amending help-seeking behaviours accordingly. Community knowledge, underpinned by individual narratives of experiences of surgery and other treatment modalities, shaped the definition of severity, with the influence of government policies and campaigns in support of hospital delivery clear in the case of pregnancy-related complications, where women favoured hospital care. Although classification by severity was the primary organizing principle for decisions about seeking help, other factors inhibited behaviour, most notably, the ability to pay for the costs of care and fears of surgical intervention.

Explanatory models were used to interpret bodily signs and sensations, to assess the severity of an individual’s condition and to guide help-seeking behaviour [23]. From our data, we identified two categories of surgical condition which invoked different patterns of behaviour. Emergency conditions were those where sensations and signs were overwhelming, i.e. Conditions that were perceived to be more severe. For these, the pathway to hospital and surgical care was clear and direct. Whilst participants acknowledged the cost, this was not seen as a barrier in situations where participants perceived that there were few other viable options to preserve someone’s life.

Government policies to reduce maternal mortality during childbirth seems to have contributed to normalization of health facility delivery and the acceptance of surgical interventions for complications that is evidenced in our narratives. In order to overcome the human resource scarcity of senior specialists within a short period of time especially in rural parts of Ethiopia, training of health officers to equip them to handle emergency obstetric-gynecological procedures has been expediated, thus making surgical care for delivery more accessible [17]. In addition, maternal care is free in public health care facilities [24], although there are still expenses women have to pay in practice. Despite these positive developments in maternal surgical care, it is clear that patriarchal norms at home and within the health systems still exert influence, meaning that women’s involvement in the decision-making process is minimal [25]. Similarly, although not mentioned by participants, the fact that injuries related to road traffic accidents have medico-legal implications in Ethiopia which require health facility-based management is likely to have influenced behaviours, encouraging people to access formal care for injuries related to this particular cause [26].

Conditions you can live with were those where signs and sensations came and went and/or were generally less severe and the aetiology more uncertain. For these conditions, the help-seeking pathway tended to be longer and more complex. This is similar to community practice in other LMICs where people have been found to explore the benefits of different types of treatment until their condition becomes severe [9, 22]. As has been found in other contexts, the approach of participants to seeking care for chronic surgical conditions was pragmatic and pluralistic, rather than being guided entirely by a rigid or discrete system of thought [27]. Our participants often started with a course of (in)action that was perceived to be low input, with a possibility of resolution, whilst understanding the potential need to escalate help-seeking at a later date. These actions shared the advantage of being low cost, locally available and familiar and therefore worth taking a risk with prior to taking more disruptive and costly measures.

For certain bearable conditions, there appeared to be an established initial preference for traditional modalities of care. Innate preference for traditional modalities and systems of thought was not explicit from our findings but may have had an indirect influence by contributing historically to common practice, which in turn has shaped community narratives about the suitability of certain conditions for particular treatments and practitioners. For example, as found in another study conducted in a tertiary hospital in Ethiopia, bone setters were thought to be better in treating a wide range of injury related conditions not caused by road traffic accidents, including fractures [28]. This view was shared by community health workers who confirmed that people visit bone setters for conditions such as dislocations, sprains and sometimes for fractures. Bone setters have built trust within the community in delivering the care for such conditions, especially when there is no associated visible bleeding. This trust may also be attributed to the fact that they refer cases which they think are beyond their capacity to health facilities, thereby helping users to construct a safe and effective pathway to care whilst minimising unnecessary costs and disruption.

Fear of surgery was one of two main barriers to initiating and completing an episode of surgical care. Fear was a prominent reason mentioned by community members in relation to delays in accessing surgical care. This was also one of the main barriers mentioned to accessing surgical care among community members in Sierra Leone and Tanzania [7, 8]. The reasons behind fear stem from the perception that undergoing surgery may leave individuals in a worse condition after the procedure, due to unexpected results of an operation or harm from mistakes by health care providers [25, 29]. Whilst apprehension about undergoing surgery is common across different healthcare and cultural settings, fear of surgery in this context is underpinned by a lack of knowledge and experience among community members and healthcare workers alike. Surgery is a relatively new concept in rural Ethiopia and there is a gap in knowledge of services available and the types of problem that can be addressed by surgery. This was perceived to adversely affect the detection and appropriate referral to a higher-level health care facility. Similar challenges were evident in a review of surgical referral systems in LMICs [19]. As a consequence, even if people do seek help rapidly, they may end up not getting the care they need because health care providers do not properly diagnose and advise them about appropriate interventions [8].

Community narratives, fuelled by individual experience, play an important role in shaping decision making and are a key driver in developing widespread trust or mistrust in a particular service, provider or treatment modality, shaping decisions about seeking care [28]. Negative narratives are as powerful as positive ones, leading to delay or to a person never accessing surgical care. For example, reports of poor outcome were reasons for not turning up for scheduled surgery in rural South Africa and Cambodia [9, 22]. Positive experiences of others drive community members to overcome fear and to access surgical care. If the cycle of positive experiences reflected in community narratives continues, community trust in a service is developed.

The other prominent barrier to surgical help-seeking was financial capacity, due to the need to not only pay direct medical fees but also indirect costs, for example, transport and hospital stay expenses. It is evident that financial constraints make a huge contribution to not accessing or delaying surgical care in LMIC [7, 8, 30, 31]. Ethiopian communities perceive surgical care to be unaffordable [25]. This encourages people to choose and explore other treatment modalities.

Our qualitative data showed that participants modified their response to surgical conditions according to their understanding of the cause and course of their problem, which guided their beliefs about viable treatment. Explanatory models were flexible: we found evidence that they evolved within the community over time, in response to government policy changes and community narratives. Individuals also altered their views about their condition over time in response to new evidence. This potential malleability has important implications for research and policy as it suggests that community-level barriers are likely to be responsive to carefully designed interventions providing there is recognition of current community knowledge and beliefs. In particular, our findings suggest three potentially impactful areas for future research. Firstly, it is clear that community narratives about treatment derived from individual experiences are powerful [32]. Therefore, quality improvement work at different levels of the health system is essential to ensure that community members who do manage to access surgical care have a positive experience [33, 34]. Linked to this, it is important that community health-workers and communities have access to information about surgery and surgical conditions that is relevant and takes into account existing explanatory models. We found that bone setters were an affordable and acceptable resource for more minor orthopedic problems, who generally encouraged clients to visit the formal health system when they believed their client’s condition was beyond their capacity. The potential for formalizing links between traditional practitioners and the health system should be explored. Research examining the potential for the integration of traditional healers for the treatment of serious mental illness has demonstrated some promising results [35]. Thirdly, it must be noted that unless financial barriers to surgical care are addressed, these are likely to undermine efforts to overcome other barriers. Finally, our experience demonstrates that conventional top-down research methods may be inherently limited in their power to understand lived experience of conditions where there are clear barriers to accessing care and community knowledge and experience of services is low. In our view, participatory methods will be necessary to fully grasp community motivations and beliefs that explain why they perceive and deal with surgical conditions as they do. Participatory Action Research has the added advantage of ensuring that participation in research leads to community driven actions to address the problems they have identified in research, which could be used to help to close the gap between unmet needs for surgical care and the character of the services provided [36].

We acknowledge that our study had some limitations. Interviews with some participants were brief. All of the shorter interviews were with community members. There were three key reasons for this: firstly, some participants lacked knowledge about their surgical conditions prior to the community survey and did not seek help and so were unable to provide detailed information about their condition. Secondly, some participants were unsure as to the nature of their health problem when they sought care, which made it difficult for them to describe their conditions to interviewers. Finally, a few participants had other health conditions, which made it difficult for interviewers to get them to talk about the surgical condition in question. Nonetheless, we believe the data presented holds sufficient information power [37] as the combination of participants included in the study were highly specific for our study aim and except for a few interviews, the dialogues were strong. Whilst shorter interviews are a methodological limitation, they are also emblematic of some of the key themes we identified in our analysis, namely: lack of knowledge of conditions requiring surgery, lack of experience of surgical care and low health literacy. Our approach to analysis evolved over time: in response to emerging themes, we moved from a discrete set of descriptive questions to an expanded analytic approach to try to understand underlying beliefs/experiences that were driving behaviours. Whilst we acknowledge alternative analytic approaches such as narrative analysis [38] may have yielded slightly different results, based on our experience of qualitative research in the same communities, we are confident that our approach was credible and appropriate, given the constraints of the wider ASSET programme.

## Conclusion

Explanatory models were found to be flexible, responsive to new evidence about what might work best in the context of limited community resources. Our findings have important implications for future research and policy, indicating that community-level barriers have the potential to be responsive to well-designed interventions which take account of local knowledge and beliefs.

### Electronic supplementary material

Below is the link to the electronic supplementary material.

Supplementary Material 1

Supplementary Material 2

Supplementary Material 3

Supplementary Material 4

Supplementary Material 5

## Data Availability

The datasets used and/or analyzed during the current study are available upon request from the corresponding author.

## Abbreviations

HEW: Health Extension Worker
IESO: Integrated Emergency Surgical Officer
LMIC: Low and Middle Income Country
SSA: Sub-Saharan Africa
