# Supplementary material for: “Problems you can live with” versus emergencies: how community members in rural Ethiopia contend with conditions requiring surgery
Source: BMC Health Serv Res. 2024 Feb 16;24:214. doi: 10.1186/s12913-024-10620-0 (PMC10874059; doi:10.1186/s12913-024-10620-0)
Supplement: Supplementary file 2 — Supplementary Material 2 [file 12913_2024_10620_MOESM2_ESM.pdf]

Topic Guide: health extension workers

1) **[surgery] What do people in this community do if a person (non-pregnant) develops severe abdominal pain?**
   Probe: Do they do anything? Who do they consult first?
   Have you been consulted for such cases? What did you do for the person? What do you think is the best thing to do for someone with severe abdominal pain?
   At what stage do people consider going to a health facility? How does that decision get made?
   What do you think are the barriers to someone with sever abdominal pain being taken to a health centre or hospital?
   What causes delay in going to the health facilities?
   How could these barriers be overcome?

2) **How about if a person has had an accident and are injured? E.g. a road traffic accident. What do people usually do?**

   Probe: Do they do anything? Who do they consult first?
   Have you been consulted by people with injuries? What did you do for the person? What do you think is the best thing to do for someone who has a severe injury?
   At what stage do people consider going to a health facility? How does that decision get made?
   What do you think are the barriers to someone with an injury being taken to a health centre or hospital? Any different from someone with severe abdominal pain?
   How could these barriers be overcome?

3) **What if people have non-emergency conditions that might benefit from surgery? Do you know of any such conditions?**
   Probe: thyroid problems? Hernias? (lumps in the groin that are reducible), Gall bladder problems? Have you heard anything about these conditions? What is your experience of people receiving surgery for these conditions?
   How acceptable do you think it is for people to have a surgical procedure for these non-emergency conditions? What barriers might stop people getting surgical care?
   Probe: financial, frightened of surgery, not aware that there is an intervention, any thing else?

4) **What do you think is needed to get the community to seek help for surgical conditions?**
   What could be the role of the health extension worker?
