# Supplementary material for: “Problems you can live with” versus emergencies: how community members in rural Ethiopia contend with conditions requiring surgery
Source: BMC Health Serv Res. 2024 Feb 16;24:214. doi: 10.1186/s12913-024-10620-0 (PMC10874059; doi:10.1186/s12913-024-10620-0)
Supplement: Supplementary file 3 — Supplementary Material 3 [file 12913_2024_10620_MOESM3_ESM.pdf]

Topic guide: persons with surgical conditions who did not access surgical care

You have told us about the health condition that affected you <*specify the type of surgical/dental condition affecting the person*>. I am going to ask you questions about that health condition.

0. [Explanatory models and preferred help-seeking]

What do you think caused <*the surgical condition*>? Anything else?
Probe: evil eye, possession, curse, bewitchment

What did you do when <*the surgical condition*> first developed? Who gave you advice about what to do? Where did you look for help first? Where did you try next?
What treatments did you try? How much did they cost? In what ways did they help you?

1. *[Barriers to accessing surgical care]*

[If the person attended a non-hospital health facility]
What made you decide to go to the health facility? How long did you wait before trying the health facility? What were the reasons for waiting?
What happened to you when you went to the health centre/health post/private clinic, etc?
How did the staff speak to you? How satisfied were you with the way that they communicated with you? Explain.
How long did you wait to be seen?

What advice were you given? What did you think of the advice that they gave you? Did you follow the advice? If not, what were the reasons that you chose not to follow the advice?

How confident were you about the skill of the health workers to manage <*surgical condition*>?
How satisfied were you with the care that you received?

Did the health workers refer you to the hospital?
If yes, did you attend?
If yes, how did you travel to the hospital? (*probe*: ambulance? Public transport? Other?). How did you pay for the transport? Were you given a referral paper? How well was the reason for referral explained to you?
How long did it take for you to go from the health centre/health post to the hospital? What happened to you when you got to the hospital?
How did the staff communicate with you? How well did they explain what was needed?
Why did you not get surgical care?
Probe: cost, quality concerns, preferring traditional methods.

If referred but did not attend, what were the reasons that you didn't go to the hospital?

[if the person did not attend a health facility]

What were the reasons that you did not go to a health facility? Any other reasons?
Probe: cost, transport availability, quality concerns, preferring traditional methods

2. *[Impact of the condition]*
   What happened to *<the surgical condition>*? Has it got better or worse over time?
   What effect has *<the surgical condition>* had on your life? Probe: work, family life, social life, how feels about self/body (self-esteem, body image).
   What has been the impact in terms of spending money on treatment? How have you paid for that? How has that affected your family?

3. *[Overcoming barriers]*
   You have told me that you didn't receive surgical care for this condition.
   What do you think could have facilitated you getting surgical care?
   What could be changed to help people access surgical care?
   Which of these is the most important to change?
