# Supplementary material for: “Problems you can live with” versus emergencies: how community members in rural Ethiopia contend with conditions requiring surgery
Source: BMC Health Serv Res. 2024 Feb 16;24:214. doi: 10.1186/s12913-024-10620-0 (PMC10874059; doi:10.1186/s12913-024-10620-0)
Supplement: Supplementary file 4 — Supplementary Material 4 [file 12913_2024_10620_MOESM4_ESM.pdf]

Topic guide: persons with surgical/dental conditions who did access care

You have told us about the health condition that affected you *<specify the type of surgical/dental condition affecting the person>*. I am going to ask you questions about that condition and the care that you have received.

[Explanatory models and preferred help-seeking]

What do you think caused *<the surgical condition>*? Anything else?

Probe: evil eye, possession, curse, bewitchment

What did you do when *<the surgical condition>* first developed? Who gave you advice about what to do? Where did you look for help first? Where did you try next?

What treatments did you try? How much did they cost? In what ways did they help you?

What made you decide to go to the health facility? How long did you wait before trying the health facility? What were the reasons for waiting?

[Delays in accessing surgical care]

[If the person attended a non-hospital health facility first]

What happened to you when you went to the health centre/health post/private clinic, etc?

How did the staff speak to you? How satisfied were you with the way that they communicated with you? Explain.

How long did you wait to be seen?

What advice were you given? What did you think of the advice that they gave you? Did you follow the advice? If not, what were the reasons that you chose not to follow the advice?

How confident were you about the skill of the health workers to manage *<surgical condition>*? How satisfied were you with the care that you received?

When you were referred to the hospital, how long did you wait before going to the hospital?

How did you travel to the hospital? (*probe*: ambulance? Public transport? Other?). How did you pay for the transport? Were you given a referral paper? How well was the reason for referral explained to you?

How long did it take for you to go from the health centre/health post to the hospital? What happened to you when you got to the hospital?

How long did you wait in the hospital before you received surgical care? What were the reasons for the wait?

[Experience of surgical care]

In the hospital, how did the staff communicate with you? How well did they explain what was needed?

How satisfied were you with the explanation about risks and benefits of the procedure?

What was the care like while you were waiting for the operation? How clean was the ward? How comfortable were you at that time? How could this have been improved?
After the operation, how was the care that you received? What could have been improved? Probe: pain relief, other reasons for discomfort
How long did you stay in hospital after the operation? When you left the hospital to go home, did you feel well enough to leave? What made you leave at that time?
What advice did the staff give you before you left? How well prepared were you to look after the wound? How much information did you have about what to do to optimise your recovery? What other information would you have liked to have been given?

What costs did you have to cover to have the operation? How difficult was it to get the money? How did you manage to get the money?

After going home after the operation, have you been reviewed in the hospital clinic?
[If not] why not? *Probe:* Not given appointment? Had appointment but didn't attend? Why?
[If yes] how satisfied were you with the clinic review? How adequate was the information you were given? How well were your questions answered?

Overall, how satisfied were you with the surgical care? What could have been improved? What could help people access surgical care more quickly?

[Impact of the condition]
Since the operation, have you had any ongoing effects of *<the surgical condition>* or the operation?
Have you looked for further treatment? If yes, where have you tried to find care? What type of treatment are you looking for? How do you hope that it will help you?
What effect has the ongoing problems with *<the surgical condition>* had on your life? Probe: work, family life, social life, how feels about self/body (self-esteem, body image).
