# Supplementary material for: “Problems you can live with” versus emergencies: how community members in rural Ethiopia contend with conditions requiring surgery
Source: BMC Health Serv Res. 2024 Feb 16;24:214. doi: 10.1186/s12913-024-10620-0 (PMC10874059; doi:10.1186/s12913-024-10620-0)
Supplement: Supplementary file 5 — Supplementary Material 5 [file 12913_2024_10620_MOESM5_ESM.pdf]

Traditional bone setters interview guide

We are interested to hear about the help you provide to people in this community.

1) *[General information on healer]*
   Can you tell me about how you became a healer?
   Please tell me about the type of work you do to help people in this community.
   What types of problems do you treat?
   How many people do you see per day?
   How do you help them?
   Probe: herbs, prayer, mediating relationship problems (talking), slaughtering sheep, baptism, fumigation, beating, exorcism, any procedures involving cutting/mending broken bones?…

2) *[Specific involvement in emergency conditions needing surgical assessment]*
   Now I would like to ask you about people who have emergency problems.
   Some examples of the kind of conditions could be:
   When somebody has a severe abdominal pain associated with vomiting
   When childbirth is prolonged
   When somebody has a serious accident/injury that leads to broken bones or internal injuries
   Urinary retention
   Failure to pass faeces and flatus
   Bleeding from any part of the body
   Loss of consciousness
   Broken bones or severe injuries

   How often do you see people who have these kinds of emergency problems?
   What approach do you take for people with these problems?
   Probe for each type of problem in turn.
   What treatment do you provide? How does the treatment work to heal them? What advice do you give? Do you monitor them over a period of time? (if so, what signs are you looking for?).
   How well do people recover from these kinds of conditions?

   Are there times when you consider referring to the health facility? What signs would make you refer? How do you think the health facility can help in that kind of situation?
   [interface with health system]
   In general, what kind of interaction do you have with the health facilities or health extension workers? How is that communication? In what ways do you think it could be improved?
   What are the areas where you think it would be possible to work with the health system? What are the areas which are for traditional/religious healing alone?
   For the emergencies described above, in what ways could you work with the health workers to improve the person's outcomes?

How would you feel about being trained to identify those cases that need an urgent referral to the hospital? What concerns would you have about this?

Do you have any other suggestions?
